# Excessive weight gain after remission of depression in a schizophrenic patient treated with risperidone: case report

**DOI:** 10.1186/1471-244X-6-37

**Published:** 2006-09-05

**Authors:** Christos G Theleritis, George N Papadimitriou, Charalabos C Papageorgiou, Dimitris G Dikeos, Vasilis Masdrakis, Constantin Kostoulas, Constantin Psarros, Constantin R Soldatos

**Affiliations:** 1 Department of Psychiatry, Athens University Medical School, Eginition Hospital, 74 Vas. Sofias Ave., Athens 11528, Greece

## Abstract

**Background:**

The use of atypical antipsychotics in schizophrenic patients has been associated with a risk of weight gain. Similarly, recovery from depression is often followed by improved appetite, greater food intake and potential increase in weight.

**Case presentation:**

A Caucasian 33-year-old schizophrenic female patient was being treated with 6 mg/day of risperidone and 15 mg/day of clorazepate. She developed depressive symptomatology and 40 mg/day of fluoxetine was gradually added to her treatment regimen for about 9 months. After the remission of depression, and the discontinuation of fluoxetine, she experienced an increase in appetite and subsequently excessive weight gain of 52 kg. Re-administration of fluoxetine did not reverse the situation. The patient developed diabetes mellitus, which was successfully controlled with metformin 1700 mg/day. The addition at first of orlistat 360 mg/day and later of topiramate 200 mg/day has helped her to lose a significant part of the weight gained (30 kg).

**Conclusion:**

The case suggests a probable association between the remission of depressive symptomatology and weight gain in a schizophrenic patient.

## Background

The beneficial effects of atypical antipsychotics on positive symptoms, negative symptoms and cognition in schizophrenia, as well as the reduced rate of extrapyramidal effects or tardive dyskinesia have led to the wide use of these drugs in clinical practice [1,2]. On the other hand their administration might lead to a marked increase in body weight in addition to metabolic abnormalities [3-13].

The efficacy of the atypical antipsychotic risperidone- a combined dopamine D2 and serotonin 5HT_2A _receptor antagonist- for both acute and maintenance therapy of schizophrenic patients is well established [1,14,15]. Regarding the effects of this drug on body weight, in a meta-analysis evaluating 10 weeks of therapy with atypical antipsychotics, the mean increase in weight was 2.10 kg [3]. Also, in a double-blind study of schizophrenic and schizoaffective patients mean weight gain after one year of risperidone therapy was 2.3 kg [16]. Furthermore, in the recent Clinical Antipsychotic Trials of Intervention Effectiveness (CATIE) study of chronic schizophrenic patients, mean increase in weight after 18 months of risperidone treatment was 0.43 ± 0.49 kg [2]; while in another involving first-episode schizophrenic patients, mean weight gain after 2 years of a risperidone regimen was 7.5 ± 0.29 kg [17]. For a more detailed review of the risk of weight gain as a result of long-term treatment with risperidone see also Ref. 12.

Risperidone, compared with olanzapine and clozapine, also seems to have a lower risk of inducing type 2 diabetes mellitus [18], although several risperidone-associated new-onset cases of diabetes have been reported [19].

Reviews of follow-up studies in patients with depression and in obese, non-depressed patients have shown that the weight-reducing effects of fluoxetine are transient [4,20] and that the long-term administration of this drug might even lead to a gain in body weight [21].

We present the case of a schizophrenic patient who, while being treated with risperidone, experienced excessive weight gain and eventually developed diabetes mellitus after the remission of depression and the discontinuation of a nine-month treatment with fluoxetine.

## Case presentation

A Caucasian 33-year-old married woman had recurrent delusions of persecution for a period of four years. Diagnosis of schizophrenia was established according to DSM-IV-TR. The severity of her psychotic symptoms was assessed using the Brief Psychiatric Rating Scale (BPRS). Family history was negative for schizophrenia and affective disorder, as well as for obesity and diabetes mellitus. At the beginning of treatment (BPRS score = 62) 6 mg/day of risperidone and 15 mg/day of clorazepate were administered; her body mass index (BMI) at the time was 29.4 (height 165 cm, weight 80 kg, baseline prolactin level = 4.5 ng/ml). After five weeks of risperidone treatment prolactin level was 18.1 ng/ml.

At the end of the first year a significant improvement in her psychotic symptoms was observed (BPRS score = 29; prolactin level = 16.1 ng/ml), but also a modest weight gain (5 kg, BMI reached 31.2). During the second year, she was fired from her job, spent more time at home and developed depressive symptomatology; 40 mg/day of fluoxetine was gradually added to her treatment regimen for about 9 months. During this time, she gained another 3 kg (BMI = 32.3; prolactin level = 13.4 ng/ml). After the remission of depression, fluoxetine was discontinued and she experienced carbohydrate cravings and "binge eating" episodes, especially during the evening. In the middle of the third year of treatment, although risperidone was gradually reduced to 2 mg/day without signs of relapse (BPRS score = 27), a dramatic increase in body weight was observed (weight 140 kg, BMI = 51.5; prolactin level = 10.7 ng/ml). All the attempts she made to reduce her weight by consulting a dietician failed. Since it was after the remission of depressive symptomatology and the discontinuation of fluoxetine that she began gaining excessive weight, fluoxetine (20 mg/day) was re-administered for few weeks but without any reduction in weight gain. This suggests that fluoxetine is not related to the patient's weight gain.

Six months later, she developed polydipsia and polyuria. Diabetes was diagnosed (her fasting blood glucose level was 170 mg/dl); thus 1700 mg/day metformin hydrochloride (an oral hypoglycaemic agent [22]) was prescribed, and after one month fasting blood glucose level was stabilized within the normal range. At the same time, in collaboration with an endocrinologist, 360 mg/day orlistat- a lipase inhibitor [23] – was administered. Her body weight was reduced to 120 kg (BMI = 44.1; prolactin level = 8.7 ng/ml) within a period of six months. During the fourth year of treatment, she was hospitalized for a six-week period in an attempt to promote further weight loss. On admission, it was decided to add 200 mg/day topiramate to her regimen, since it has been reported that topiramate is an anticonvulsant with mood stabilizing and weight loss properties [24]. This helped her to lose a further 10 kg in a six-month period (BMI = 40.4; prolactin levels = 7.2 ng/ml).

The patient never had any abnormalities regarding thyroid function or her menstrual cycle (the levels of reproductive hormones were within normal range) during the four years of treatment. Prolactin levels were not elevated throughout the treatment with risperidone (range of prolactin levels in our patient under risperidone treatment: 7.2–18.1 ng/ml, normal prolactin levels: 2.5–26.5 ng/ml). It is worth noting that the patient complied well with pharmacotherapy.

## Conclusion

This is a report on a case of a schizophrenic patient who experienced excessive weight gain and diabetes mellitus while being treated with risperidone.

It has been suggested that the average increase in body weight under risperidone treatment is approximately 2–2.5 kg, both after 10 weeks and after one year [3,16]; while after 18 months in chronic schizophrenics and 2 years in first-episode schizophrenics mean weight gain is 0.43 ± 0.49 kg and 7.5 ± 0.29 kg respectively [2,17]. The risperidone-induced weight gain, as a percentage of baseline body weight, is more pronounced among pre-adolescents and decreases with advancing age and especially in adults over the age of 65. In youths and middle-aged adults, weight increase related to risperidone is greatest during the first few months of treatment, but in certain cases can persist beyond one year [25].

In addition to medication type and age, other factors can influence antipsychotic-induced weight gain, such as lower BMI and extended duration of treatment [3,12,25-27]. Studies of olanzapine, risperidone, and quetiapine have clearly documented that adults whose pre-treatment BMI is less than 23 have substantially more drug-induced weight gain than those whose baseline BMI exceeds 27 [25]. Although our patient had a BMI of 29.4 when she started treatment with risperidone, after 2 1/2 years she had a substantial further increase in BMI to 51.5 and the development of diabetes mellitus. Patients under treatment with atypical antipsychotics who have a higher baseline BMI are at increased risk of diabetes, compared to patients with a lower BMI [6].

Recovery from depression and from psychotic symptoms is often associated with improved appetite and better social functioning, which could lead to greater food intake and potential weight gain [8,20,27]. Serotonin has been implicated in the control of eating behavior and body weight. Stimulants of serotoninergic transmission reduce food intake and weight gain and increase energy expenditure both in animals and in humans [28]. Fluoxetine was reported to have anorectic properties [20] and to be possibly efficacious in reducing binge-eating frequency and severity in these patients, although both properties of this drug have been challenged [4,21,29].

Fluoxetine (20 mg/day) was administered again, in the middle of the third year of treatment, for a period of a few weeks; but the situation regarding weight gain did not improve and the drug was once more discontinued.

The administration of orlistat, helped the patient to lose 20 kg of body weight. It is worth noting that this drug has not been systematically studied in patients with mental illness or antipsychotic-induced weight gain [24]. The addition of metformin to the patient regimen did not result in weight loss. These results are in accordance with a pilot cross-over study on five female schizophrenic patients in which this drug did not lead to significant weight loss compared to placebo [30]. Topiramate has been suggested as a potential weight-reducing agent when it is used as an adjunct to an existing mood stabilizer or antipsychotic therapy, or when it replaces a mood stabilizer [24]. The adition of topiramate 200 mg/day to orlistat led to a further reduction of body weight by 10 kg during the fourth year of treatment with risperidone.

The mechanisms responsible for weight gain in schizophrenic patients are not fully understood. Weight gain is due to either increased energy intake, decreased energy expenditure or both, and may also depend on complex interactions between various drugs [9]. Moreover, factors affecting the decision to eat are complex and involve environmental, cognitive, emotional and behavioral elements [31].

In schizophrenic patients diets tend to be characterized by a lack of fruit, vegetables, fibre, an excess of calories and saturated fat, while physical activity levels appear to be very low [32]. It is proposed that these people may benefit by participating in lifestyle management programs early in the course of the illness [9,11,32]. Our patient presented increased energy intake, decreased energy expenditure and lack of physical activity which possibly contributed to the excessive increase in weight.

The observation of excessive weight gain in a patient treated with antipsychotics after the remission of depression suggests the need for caution. However, further observations of patients in the same condition should be carried out in order to clarify whether our findings are replicable.

## Abbreviations

D_2 _= dopamine-2

5HT_2A _= serotonin -2A

BMI = body mass index

## Competing interests

The author(s) declare that they have no competing interests.

## Authors' contributions

CGT, GNP, CCP, CP, VM, CK conceptualized and followed up the patient during the four years of her treatment. CGT, GNP and DGD did literature survey and wrote the report. CRS took part in the scientific discussion and in finalizing the manuscript. All the authors read and approved the final document.

## Pre-publication history

The pre-publication history for this paper can be accessed here:


